# Effect and mechanisms of exercise for complex regional pain syndrome

**DOI:** 10.3389/fnmol.2023.1167166

**Published:** 2023-05-03

**Authors:** Tian-Shu Li, Rui Wang, Xuan Su, Xue-Qiang Wang

**Affiliations:** ^1^Department of Sport Rehabilitation, Shanghai University of Sport, Shanghai, China; ^2^Department of Rehabilitation Medicine, Shanghai Fourth People’s Hospital, School of Medicine, Tongji University, Shanghai, China; ^3^Shanghai Shangti Orthopaedic Hospital, Department of Rehabilitation Medicine, Shanghai, China

**Keywords:** complex regional pain syndrome, exercise, pain, analgesic effect, analgesic mechanisms

## Abstract

Complex regional pain syndrome characterized by severe pain and dysfunction seriously affects patients’ quality of life. Exercise therapy is gaining attention because it can effectively relieve pain and improve physical function. Based on the previous studies, this article summarized the effectiveness and underlying mechanisms of exercise interventions for complex regional pain syndrome, and described the gradual multistage exercise program. Exercises suitable for patients with complex regional pain syndrome mainly include graded motor imagery, mirror therapy, progressive stress loading training, and progressive aerobic training. In general, exercise training for patients with complex regional pain syndrome not only alleviates pain but also improves physical function and positive mental status. The underlying mechanisms of exercise interventions for complex regional pain syndrome include the remodeling of abnormal central and peripheral nervous system, the regulation of vasodilation and adrenaline levels, the release of endogenous opioids, and the increased anti-inflammatory cytokines. This article provided a clear explanation and summary of the research on exercise for complex regional pain syndrome. In the future, more high-quality studies with sufficient sample sizes may provide more exercise regimens and better evidence of efficacy.

## 1. Introduction

Complex regional pain syndrome (CRPS) is a chronic pain condition characterized by autonomic and inflammatory features and usually affects the distal limb (Bruehl, 2015; Smart et al., 2016; Goebel et al., 2019). The pathogenesis of this disorder is not fully understood, but it is usually triggered by a limb injury, such as trauma or surgery with or without specific nerve injuries. CRPS may develop after major trauma, minor injury, or surgery, and progress from self-limited and mild symptoms to chronic disease (Urits et al., 2018). Female and individuals with upper extremity injuries or suffered from a high-energy trauma are at a higher risk of developing CRPS (de Mos et al., 2007; Petersen et al., 2018). Patients with CRPS usually suffer from skin temperature changes allodynia, hyperalgesia, oedema, and impaired motor function (Petersen et al., 2018). In many instances, the development of CRPS is debilitating and severely reducing patients’ life quality, placing an enormous burden on their families (van Velzen et al., 2014). Although some symptoms of CRPS may get better spontaneously, aggressive treatment should not be delayed because progressive deterioration of symptoms are related to poor prognosis (Bean et al., 2014; Urits et al., 2018). Appropriate management may hasten the recovery of CRPS (Bean et al., 2016). The common treatment of CRPS is symptomatic including physical therapies, occupational therapies, psychological therapies, anti-inflammatories, neuropathic pain medications, and interventional procedures (Urits et al., 2018; Harden et al., 2022). Exercise therapy is an effective and affordable component of physical therapy in the management of CRPS (Smidt et al., 2005; McCormick et al., 2015). Previous studies have reported that exercise can reduce pain and edema volume and improve overall function in daily life activities for patients with CRPS (Sherry et al., 1999; McCormick et al., 2015; Sezgin Ozcan et al., 2019; Harden et al., 2022). Exercise can also reduce negative mood and improve patients’ general well-being. However, the potential therapeutic mechanism of exercise intervention for CRPS is lacking. This review summarizes the effectiveness of exercise on CRPS and comprehensively discusses the underlying mechanisms behind it to help researchers better understand the progress in this area.

## 2. Effect of exercise on CRPS

For patients with CRPS, starting exercise rehabilitation early provides the best probability of a good outcome and minimizes distress according to the practical guidelines (Goebel et al., 2019; Harden et al., 2022). The guideline for the management of CRPS from the European Pain Federation Working Group in 2019 recommended that patients with CRPS take appropriate, generally gentle, and graded exercises as soon as possible in the presence of pain and avoid immobilization of the CRPS limb (Goebel et al., 2019). Another guideline published in 2022 recommended that the principle of functional restoration for CRPS is based on a gradual and steady advancement: from activation of premotor and primary motor cortices to very gentle active movements, to stress loading and aerobic training, then to movements that comprise more active load bearing, and finally to vocational rehabilitation, thereby preparing to resume patients’ daily life and work (Harden et al., 2022).

Graded motor imagery consists of limb laterality recognition, motor imagery, and consecutive mirror therapy, which is designed specifically for patients with longstanding CRPS to shorten the prognostic course of CRPS (Moseley, 2004). Mirror therapy is among the most effective treatments to improve functional impairment for patients with acute CRPS (Cacchio et al., 2009a). Moseley (2006) reported that 6 weeks of graded motor imagery training significantly improved pain severity and functional impairment in patients with CRPS, and the effect was maintained at 6 months of follow-up. Mirror therapy and graded motor imagery can significantly relieve pain and improve motor control by helping the patients focus on the affected extremity, increase perceived ownership of that extremity, reduce kinesiophobia, and correct the mismatch between the motor and sensory systems (Cacchio et al., 2009a; Mccabe, 2011). Gentle active movements like initiating from active range of motion are performed to manage edema and conduct preliminary desensitization (Stanton-Hicks et al., 1998). A case reported that a female patient with CRPS-type 1 had pain and edema relief and function improvement after 20 days of range of motion exercise (Oh et al., 2019). Stress loading and aerobic training are also recommended. Although stress loading may initially increase symptoms in the affected extremities, pain and swelling usually decrease after several days (Harden et al., 2022). Watson and Carlson (1987) reported that 3 years of stress loading training for 41 CRPS patients remarkably improved pain and dysfunction, enhanced muscle strength, and greatly return to daily activities. Pain exposure physical therapy involves a progressive loading exercise program and management of pain avoidance behavior. Two studies found that 4 weeks to 3 months of pain exposure physical therapy for patients with CRPS-type 1 substantially reduced pain and functional limitations (van de Meent et al., 2011; Barnhoorn et al., 2015). Aerobic training contributes to manage edema, optimize range of motion, and improve circulation. A single-blind randomized controlled trial concluded that 4 weeks of aerobic exercise significantly reduced the signs and symptoms of CRPS-type 1 compared with conventional therapy (Topcuoglu et al., 2015). In addition, aquatic therapy is especially valuable for patients with CRPS (Harden et al., 2022). The gentle compressive force provided by hydrostatic pressure gentle around the extremity may reduce the widespread edema, dampen sympathetic nerve activity, and finally relieve pain (Yamazaki et al., 2000; Hall et al., 2008; Harden et al., 2022). Aquatic therapy is also beneficial to reduce extremity weight loading, and buoyancy may facilitate early recovery of functional activities (Saquetto et al., 2019; Harden et al., 2022). Sezgin Ozcan et al. (2019) reported that patients with CRPS-type 1 achieved better improvements on neuropathic pain and edema volume after performing active range of motion exercises in the water compared with the conventional rehabilitation program. Details of studies on exercise interventions for CRPS are presented in Table 1.

**Table 1:** General characteristics of clinical studies on exercise for complex regional pain syndrome.

First author, year	Participants				Exercise intervention		Adjuvant therapies	Outcomes	Results
	Sample size (women/men)	Mean age	Type	Course	Type	Duration			
Saha et al. (2021)	38 (1/2)	EG:59.7 CG:57.4	CRPS-type 1 of the unilateral upper extremity	Time since stroke: EG:13.27 mon CG:13.47 mon	Exercise administered in front of mirror (neurodevelopmental facilitation techniques, range of motion exercises, stretching and task training)	30 min/day, 5 days/week, 4 weeks	Continue regular medications	(1) Pain: Neuropathic pain rating scale (2) Oedema: wide tape measure (3) Functional status: Functional independence measure	(1) Achieving 35% improvement at post-treatment (−2.14pts) and 43% improvement (−2.6pts) at follow-up (*p* < 0.05), significantly better than the CG (*p* < 0.05). (2) Significant improvement at post-treatment (−2.53 cm) and follow-up (−3.07 cm), better than the CG (*p* < 0.05). (3) Significant improvement at post-treatment (−15.86) and follow-up (−19.6), better than the CG (*p* < 0.05).
Sezgin Ozcan et al. (2019)	30 (3/2)	EG:62.6 CG:63.5	CRPS-type 1 of the unilateral upper extremity	Time since stroke: EG: 4 mon CG:5 mon	Active range of motion exercises in water	20 min/session, 5 sessions /week, 15 sessions	NR	(1) Pain: VAS at rest and with activity (2) Neuropathic Pain: The painDETECT questionnaire (3) Oedema: Volumetric measurements (4) Motor recovery: Brunnstrom motor recovery stages (5) Functional status: Functional independence measure	(1) Achieving 33.3% improvement at rest (−2pts) and 37.5% improvement during activity (−3pts) after the intervention (*p* < 0.05), but no significant intergroup difference. (2) Significant improvement after the intervention, better than the CG (*p* < 0.05). (3) Significant improvement (−40 mm) after the intervention, better than the CG (*p* < 0.05) (4) Significant improvement after the intervention (*p* < 0.05), but no significant intergroup difference. (5) Significant improvement after the intervention (*p* < 0.05), but no significant intergroup difference.
Barnhoorn et al. (2015)	56 (45/11)	EG:43.7 CG:43.1	CRPS-type 1	7.2 mon	Pain exposure physical therapy (Progressive-loading exercise and management of pain avoidance behavior)	40 min/session, 5 sessions	No drugs during intervention	(1) Pain: VAS (2) Joint mobility: active range of motion (3) Disability: Pain disability index	(1) Achieving 43% improvement (−2.66pts) after 9 months (*p* < 0.05), but no significant intergroup difference. (2) Significant improvement after the intervention, better than the CG (*p* < 0.05). (3) Significant improvement after the intervention (*p* < 0.05), but no significant intergroup difference.
Topcuoglu et al. (2015)	52 (9/11)	EG:66.0 CG:67.5	CRPS-type 1 on the hemiplegic side	Time since stroke: EG:2.5 mon CG:2.7 mon	Upper extremity aerobic exercise	30 min/day, 5 days/week, 4 weeks	(1) Medications: Non-steroidal anti-inflammatory medication, diclofenac Na and paracetamol (2) Others: Transcutaneous electrical nerve stimulation, (3) cold-pack, massage, and contrast baths	(1) Pain: Neuropathic pain rating scale (2) Quality of life: Nottingham health profile (3) Mood: Beck depression scale	(1) Achieving 89.9% improvement after the intervention (*p* < 0.05), significantly better than the CG (*p* < 0.05). (2) Significantly better than the CG (p < 0.05). (3) Significantly better than the CG (p < 0.05).
Van De Meent et al. (2011)	20 (NR)	38.9	CRPS-type 1	6.5 mon	Pain exposure physical therapy (Progressive-loading exercise and management of pain avoidance behavior)	1 h, a maximum of 6 sessions, 4 weeks-3 months	No drugs during intervention	(1) Maximal pain: VAS (2) Total pain intensity: McGill Pain Questionnaire (3) Grip strength (4) Disability (5) Walking Capacity: 10-meter walking speed (6) Quality of life: Short- form 36	(1) Achieving 35% improvement at post-treatment (−20pts) and 57% improvement (−33.1pts) at follow-up (*p* < 0.05), compared to the baseline (*p* < 0.05). (2) Significant improvement at post-treatment (27%) and follow-up (48%) compared to the baseline (*p* < 0.05). (3) Significant improvement with a smaller difference between sides by 52% (*p* < 0.05). (4) Achieving 60% improvement at post-treatment compared to the baseline (*p* < 0.05). (5) Achieving 29% improvement at post-treatment compared to the baseline (*p* < 0.05). (6) Achieving 26.9% improvement at post-treatment compared to the baseline (*p* < 0.05).
Cacchio et al. (2009a)	48 (13/11)	EG:57.9 CG:58.8	CRPS-type 1 of the unilateral upper extremity	EG:2.8 mon CG:2.6 mon	Mirror therapy	30 min (for the first 2 weeks) and 1 h (for the last 2 weeks), 4 weeks	NR	(1) Pain: VAS (rest, movement and tactile allodynia) (2) Function: Wolf Motor Function Test (3) Disability: Motor Activity Log	(1) Achieving 43% improvement at rest (−3.3pts), 41% on movement (−3.6pts), and 44% on allodynia (−3pts) after the intervention (*p* < 0.05); achieving 38% improvement at rest (−2.9pts), 45% on movement (−3.9pts), and 49% on allodynia (−3.3pts) at follow-up (*p* < 0.05); significantly better than the CG (*p* < 0.05). (2) Significant improvement after the intervention and at follow-up (*p* < 0.001), better than the CG (*p* < 0.01). (3) Significant improvement after the intervention and at follow-up (*p* < 0.001), better than the CG (*p* < 0.01).
Moseley (2004)	13 (9/4)	EG:35.0 CG:38.0	CRPS-type 1 of the unilateral upper extremity	Time since stroke: EG:12 mon CG:15 mon	Graded motor imagery training (including limb laterality recognition, motor imagery, and mirror therapy)	2 weeks for limb laterality recognition, 2 weeks for motor imagery, 2 weeks for mirror therapy	Continue regular medications	(1) Pain: Neuropathic pain scale (2) Oedema: The girth of the base of the fingers	(1) Significantly improvement compared to the baseline (*p* < 0.001). (2) 50% of patients improved and no longer fulfilled the diagnostic criteria for CRPS after 6 weeks training.
McCabe et al. (2003)	8 (5/3)	33.0	CRPS-type 1	Early stage: 3 people Intermediate stage: 2 people Chronic stage: 3 people	Mirror visual feedback	Every day, 6 weeks	Opioid, non-steroidal anti-inflammatory drug	(1) Pain: VAS (2) Vasomotor: Infrared thermography	(1) Achieving 100% pain reduction in the early CRPS after the intervention, but not obvious in the intermediate CRPS and no change in the chronic CRPS. (2) Reversal of vasomotor changes and normalization of function in the early and intermediate CRPS, but no change in the chronic CRPS.
Pervane Vural et al. (2016)	30 (13/9)	EG:68.9CG:61.4	CRPS-type 1 of the upper extremity	Time since stroke: EG: 4 mon CG:6 mon	Mirror therapy	30 min/session, 5 sessions /week, 4 weeks	NR	(1) Pain: VAS (2) Motor recovery: Brunnstrom recovery stages (3) Motor function: the Fugl-Meyer Assessment (4) Functional status: Functional independence measure-motor	(1) Achieving 50% improvement (−3pts) after the intervention (*p* < 0.05), better than the CG (*p* < 0.01). (2) Significant improvement after the intervention (*p* < 0.05). (3) Significant improvement after the intervention (*p* < 0.05). (4) Significant improvement after the intervention (*p* < 0.05), better than the CG (*p* < 0.05).
Cacchio et al. (2009b)	24 (13/11)	62.0	CRPS-type 1 of a paretic arm	Time since stroke:14 mon	Mirror therapy	30 min/day, every day, 4 weeks	No drugs during intervention	(1) Pain: VAS (2) brush-induced allodynia (3) Function: Wolf motor-function test (4) Oedema	(1) Significant pain reduction (−51pts) in 88% of patients after the intervention (*p* < 0.01). (2) Improved after the intervention. (3) Improved after the intervention. (4) Improved after the intervention.
Oh et al. (2019)	1 (One female)	19.0	CRPS-type 1 of the right upper extremity	3 mon	Passive range of motion exercise	under sedation (30 min, 2 days) and without sedation (30 min, twice daily, 18 days)	Midazolam	(1) Pain: Neuropathic pain rating scale (2) Oedema: the circumference of hand, wrist, and elbow (3) Strength: Manual muscle test (4) Function: Jebsen–Taylor hand function test	(1) Achieving 33% improvement (−7pts) after intervention (*p* < 0.05). (2) Decreased 5 cm at hand, 2 cm at wrist, and 6 cm at elbow. (3) Significant improvement after the intervention (*p* < 0.05). (4) The score increased from 0 to 43.
Watson and Carlson (1987)	52 (25/27)	53.0	CRPS-type 1	5.4 mon	Active stress loading program (active traction and compression exercises)	Every day, 3 years	No drugs during intervention	(1) Pain: VAS (2) Joint mobility: active range of motion (3) Grip strength (4) Daily activity level	(1) Achieving 65% improvement (−5pts) in 88% of patients after the intervention (*p* < 0.05). (2) Significant improvement in 95% of patients (*p* < 0.05). (3) All patients showed an improvement. (4) 95% of patients returned to their normal activities.
Sherry et al. (1999)	103 (87/16)	13.0	CRPS-type 1	2 mon	An intensive exercise program (aerobic exercise training, functional activities, and aquatic aerobic training)	4 h/day, 14 days	Acetaminophen	(1) Pain: VAS (2) Dysfunction: self-report and observation	(1) Achieving 99% improvement(−75pts) in 92% of patients at follow-up. (2) 92% of patients had full functional recovery at follow-up.
Wu et al. (1999)	26 (NR)	NR	CRPS-type 1	Late stage	Qigong exercise	40 min/session, 6 sessions for 3 weeks	NR	(1) Pain: VAS (2) Mood: Anxiety assessment	(1) 82% of patients reduced pain after the first training session, and 91% reduced pain after the last training session in the EG. (2) Significant improvement, better than the CG (*p* < 0.05).
Pleger et al. (2005)	6 (NR)	NR	CRPS-type 1 affected the whole hand	NR	Graded desensitization and motor tasks	15–30 min/session, 2–3 sessions, 3–4 days/week, 6 months	Continue regular medications (celecoxib, valdecoxib, gabapentin)	(1) Pain: Numeric rating scale (2) Functional magnetic resonance imaging of the brain (3) Tactile acuity: Two-point discrimination thresholds	(1) Achieving 76% improvement (−3.8pts) after the intervention (*p* < 0.05). (2) Significant regaining of cortical map size in contralateral primary and secondary somatosensory cortex compared to the baseline (*p* < 0.05). (3) Significant restoration of the impaired tactile discrimination (*p* < 0.05).

EG, Experimental Group; CG, Control Group; CRPS, complex regional pain syndrome; VAS, Visual Analog Scale; NR, not reported.

## 3. Underlying therapeutic mechanisms of exercise on CRPS

**Figure 1:**
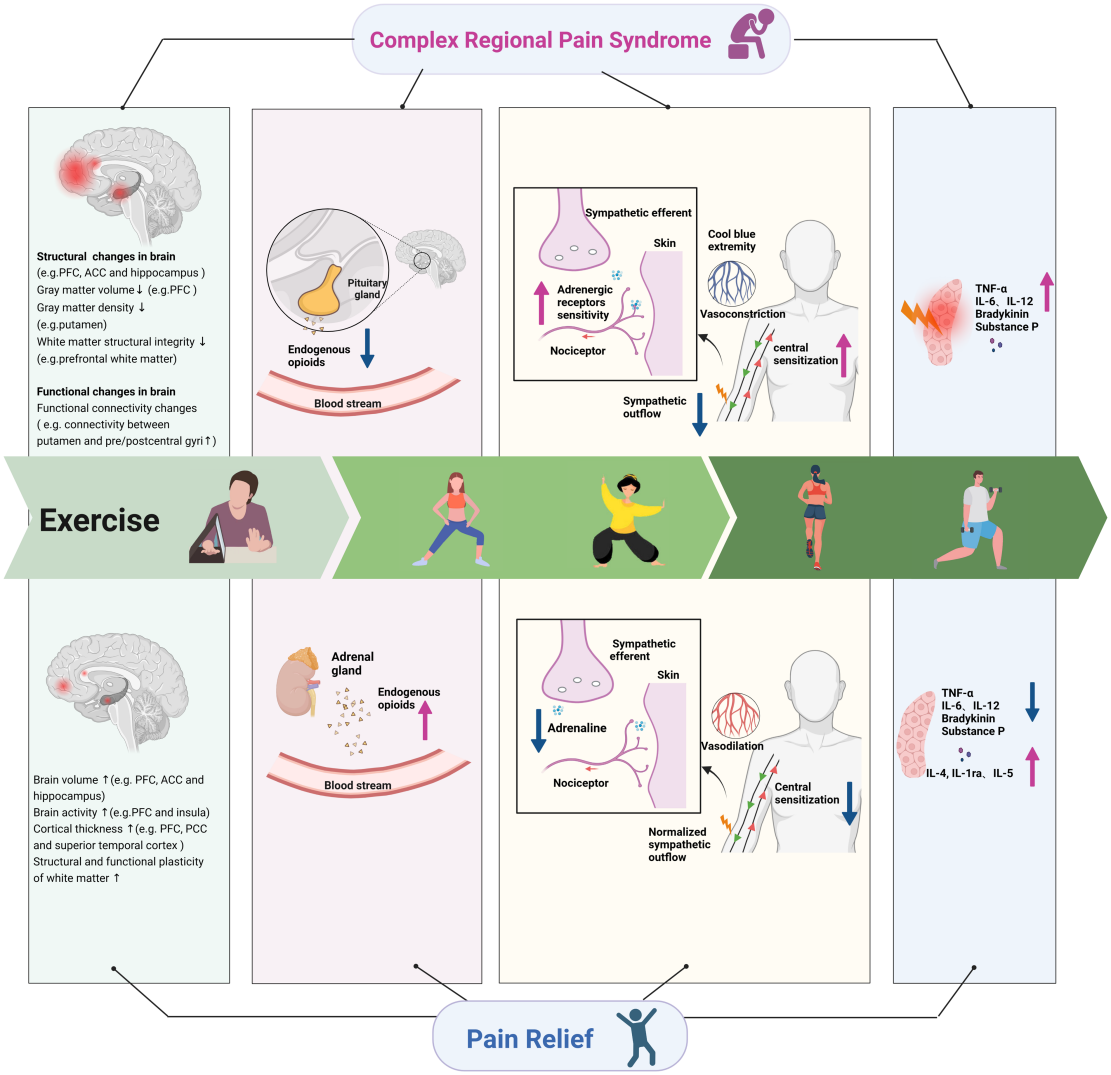
Underlying mechanisms of exercise on complex regional pain syndrome. ACC, anterior cingulate cortex; PFC, prefrontal cortex; PCC, posterior cingulate cortex; TNF, tumor necrosis factor; IL, interleukin; 5-HT, 5-hydroxytryptamine.

Abnormal remodeling of the central nervous system is common in chronic pain, and CRPS is often secondary to damage of the nervous system, such as stroke (Baliki et al., 2011; Yang and Chang, 2019; Martins et al., 2022). When nervous system injury or abnormal nervous system function (central sensory signal amplification) occurs, the spinal cord and brain regions involved in pain processing can undergo great changes (Costigan et al., 2009; Coppieters et al., 2016). Patients with CRPS often present with extensive hypersensitivity to pain, decreased pain threshold, and increased duration of pain (Mendell, 2014; Birklein et al., 2015). Exercise can induce hypoalgesia (Rice et al., 2019; Wewege and Jones, 2021), and the analgesic effect of proximal motor site and distal nonmotor sites induced by aerobic exercise showed an overall effect (Zheng et al., 2021). Therefore, the analgesic effect of exercise on patients with CRPS may also involve a combination of various mechanisms (Figure 1).

### 3.1. Improvement of sensitization of the central and peripheral nervous systems

CRPS pain and punctuate mechanical hyperalgesia can predict the cortical reorganization in the central nervous system (Maihöfner et al., 2003). Various studies have confirmed that exercise can regulate cortical reorganization (Carey et al., 2002; Pleger et al., 2005; Laible et al., 2012). The magnetoencephalography showed extensive reorganization of the primary somatosensory cortex contralateral to the affected side of CRPS, and pain reduction in CRPS correlated with recovery from cortical reorganization (Maihöfner et al., 2003, 2004). Pleger et al. (2005) concluded that one to six months of behavioral treatments consisting of graded sensorimotor retuning resulted in a sustained reduction in pain intensity for CRPS patients with intractable pain, which was accompanied by recovery of the impaired tactile discrimination and cortical map size in contralateral somatosensory cortexes. Brain functional magnetic resonance imaging in patients with CRPS revealed structural or functional changes in thalamus, hippocampus, amygdala, somatosensory cortex, primary motor cortex, prefrontal cortex (PFC), anterior cingulate cortex (ACC), insula cortex, and other brain areas (Bolwerk et al., 2013; Erpelding et al., 2016) involved in pain perception (Izquierdo-Alventosa et al., 2020). Bolwerk et al. (2013) found that the functional connectivity of sensorimotor cortex and intraparietal sulcus was more diffuse within other brain regions in CRPS patients. Physical exercise has been proved to induce structural plasticity in the human brain (Colcombe et al., 2006). Rogge et al. (2018) found that balance training for 12 weeks in healthy adults increased the cortical thickness in the superior frontal sulcus, the superior temporal cortex, the posterior cingulate cortex, the visual association cortices, and the precentral gyrus. Several studies revealed decreased gray matter volume in the PFC regions in CRPS patients compared with healthy subjects, and the atrophy in the PFC was correlated with the duration and intensity of CRPS pain (Geha et al., 2008; Barad et al., 2014; Lee et al., 2015). Exercise increased brain activities in the PFC and the anterior insula (Ellingson et al., 2016), and long-term brisk walking can increase the gray matter volume of PFC and ACC in healthy old people (Colcombe et al., 2006). The white matter integrity is widely affected in patients with CRPS (Geha et al., 2008; Hotta et al., 2017). The structural integrity of the prefrontal white matter in CRPS patients was lower than in healthy people due to the high degree of pain catastrophizing (Im et al., 2021). Mendez Colmenares et al. (2021) observed positive changes in the myelinating regions after 6 months of aerobic walking and dance intervention in healthy adults, thereby signifying that aerobic exercise training can induce the plasticity of white matter regions. Motor skill training activates neurons to release neurotransmitters, which promote the formation of mature myelinated oligodendrocytes (Gibson et al., 2014; Guo et al., 2020). In a mice model of chronic incomplete spinal cord injury, exercise induced oligodendrogenesis, increased axonal oligodendrocyte interactions, promoted white matter plasticity, and thus reduced hyperalgesia and neuropathic pain behavior (Faw et al., 2021).

CRPS pain is also related to gray matter hypertrophy in the left amygdala, left posterior hippocampus, and right hypothalamus (Barad et al., 2014). The two regions are generally associated with emotional intensity encoding and limbic reward processing (Rolls, 2015; Corbett et al., 2020). Aerobic training was showed to increase hippocampal volume in young healthy adults and old people without dementia (Erickson et al., 2011; Rogge et al., 2018). There was a positive correlation between increasing fitness levels and changes in the hippocampal perfusion after 3 months of intervention (Maass et al., 2016). Another study reported that mind–body exercise increased gray matter volume in the right hippocampus and in the bilateral ACC in people with mild cognitive impairment (Tao et al., 2019). Patients with CRPS were accompanied with bilateral decreases in gray matter density in the putamen and functional connectivity changes among the putamen, cerebellum and pre/postcentral gyri (Azqueta-Gavaldon et al., 2020). These abnormalities affected pain processing and implicated movement disorders. Nagamatsu et al. (2016) reported that healthy individuals with the greatest improvement in mobility had the greatest left putamen volume retention after 12 months of training.

### 3.2. Regulation of vasodilation and adrenaline levels

CRPS-type 1, known as reflex sympathetic dystrophy, is a sympathetically mediated peripheral pain condition. After the injury occurred, nociceptive fibers in the injured area initiate to express adrenergic receptors. Reduced sympathetic outflow following peripheral nerve injury leads to compensatory up-regulation of local adrenergic receptor sensitivity in the affected limb (Raja et al., 1992; Bruehl, 2010). This up-regulation may lead to exaggerated catecholamine responsiveness, which leads to excessive vasoconstriction, thereby causing the classic symptoms of cool blue extremity in chronic CRPS. Topcuoglu et al. (2015) suggested that an endothelium-related vasodilation mechanism caused by exercise could alleviate the situation. Mortensen et al. (Mortensen et al., 2014) found that 8 weeks of exercise training reduced the vasoconstrictor response to sympathetic nerve activity and improve the ability to override sympathetic vasoconstrictor activity. Another study indicated that physical training in patients with chronic heart failure restored endothelial dysfunction by enhanced endothelial release of nitric oxide to coordinate tissue perfusion (Hornig et al., 1996). In addition, catecholamine-induced nociceptive firing may promote central sensitization by maintaining elevated peripheral nociceptive input (Gracely et al., 1992). Central sensitization causes an increase in pain and catecholamine release that further causes a vicious cycle (Gracely et al., 1992). Therefore, a decrease in epinephrine levels caused by exercise is possibly beneficial (Kiilavuori et al., 1999). Animal experiments showed a decrease in vascular sensitivity after immediate exercise and long-term exercise training (Izawa et al., 1996), and exercise normalized sympathetic outflow by central antioxidant mechanisms (Gao et al., 2007). Negative emotions are also associated with increased catecholamine release (Charney et al., 1990; Light et al., 1998; Bruehl, 2010). Severe depressive symptoms were related to an elevated level of plasma epinephrine (Harden et al., 2004). Exercise may indirectly regulate the release of catecholamines by improving mood, and finally achieve pain relief in CRPS.

### 3.3. Release of endogenous opioids

Endogenous opioids, such as endorphin and enkephalin, may play a vital role in exercise-induced reduction of CRPS pain (Misra et al., 2017). A study suggested that failed opioid modulation in the regional sympathetic ganglia may trigger or contribute to CRPS-type 1 accompanied by intense pain (Hannington-Kiff, 1991). One explanation for this mechanism is the characteristic of CRPS that complicates minor injuries, indicating that minor injury fails to start or maintain adequate opioid modulation in the regional sympathetic ganglia. The alternative explanation is the developing tolerance to regionally increase opioid activity (Hannington-Kiff, 1991). A study showed that immunoreactive β-endorphin levels in peripheral blood mononuclear cells were significantly lower in the CRPS patients (Takahashi et al., 2000). These findings suggest that regional increases in endogenous opioids might be effective. Adequate exercise intensity and duration were demonstrated to increase circulating β-endorphin and enkephalin levels (Goldfarb et al., 1990). Physical therapy combined with active use of the affected limb may offer a conducive way to sustain regional opioid modulation and safeguard the limb from CRPS-type 1 (Hannington-Kiff, 1991). The results of Pierce et al. showed a remarkable increase in β-endorphin levels after 45 min of high-intensity aerobic exercise (Pierce et al., 1993). Plasma beta-endorphin concentration was elevated after light load blood flow restriction resistance exercise (Hughes and Patterson, 2020). Another study, which included 59 healthy women, found a significant increase in plasma proenkephalin peptide F with acute exercise, and this effect was greater in the combination of strength and endurance training group (DuPont et al., 2017). Furthermore, in a mice model of CRPS-type 1, Martins et al. found that swimming exercise decreased allodynia, and the antiallodynic effect induced by exercise was reversed by the pretreatment with a nonselective opioid receptor antagonist (naloxone), confirming the role of the endogenous opioid in the antiallodynic effect of exercise (Martins et al., 2013). Chromaffin cells of the adrenal medulla are thought to be rich source of endogenous opioids. Results from a study showed that the bilateral adrenalectomy in mice suppressed the analgesia effects of high intensity swimming exercise, suggesting that endogenous opioids released by adrenal glands may conduce to the analgesia effect induced by exercise (Mazzardo-Martins et al., 2010).

### 3.4. Increased levels of anti-inflammatory cytokines

Persistent inflammatory activities in patients with CRPS lead to visible signs, such as edema, severe pain, temperature increase, and hyperalgesia. Pain catastrophizing in patients with CRPS is related to elevated pro-inflammatory cytokine activity in reaction to painful stimuli (Edwards et al., 2008). Cytokines cause pain and hyperalgesia through the sensitization of nociceptors and release abundant neuropeptides (Birklein et al., 2015). In the samples of CRPS patients’ serum and cerebrospinal fluid, increased levels of proinflammatory cytokines (interleukin–6, interleukin-12, tumor necrosis factor alpha receptors), decreased levels of anti-inflammatory cytokine, and increased levels of neuropeptides (bradykinin, calcitonin substance P and gene-related peptide) were found (Marinus et al., 2011). Exercise may participate in inhibiting regulated neuropeptide signaling and inflammatory mediator expression and reversing nociceptive sensitization. A study in the mouse model of CRPS found that 4 weeks of running wheel exercise can reverse the upregulation of neuropeptide and inflammatory mediator expression (Shi et al., 2018). However, the pain behaviors recurred when exercise stopped for 2 weeks, this nociceptive sensitization was related to increased neuropeptide levels, interleukin-6, and nerve growth factor expression (Shi et al., 2018). Evidence from animal and clinical studies showed that exercise can increase anti-inflammatory cytokines and reduce the levels of pro-inflammatory cytokines (Kohut et al., 2006; Tsai et al., 2017; Paolucci et al., 2018). Six weeks of high-intensity interval training decreased the level of tumor necrosis factor alpha in healthy adults (Paolucci et al., 2018). Two weeks of treadmill exercise improved neuropathic pain (mechanical hyperalgesia and avoidance behavior) in mice. Meanwhile treadmill exercise increased the levels of M2 macrophages which secretes anti-inflammatory cytokines, decreased the levels of M1 macrophages which secretes proinflammatory cytokines, and increased anti-inflammatory cytokine concentrations (interleukin-4, interleukin-1ra, and interleukin-5; Bobinski et al., 2018). Therefore, exercise modulates the immune system to promote healing and analgesia (Sluka et al., 2018; Simpson et al., 2021).

## 4. Conclusion

The exercise rehabilitation for patients with CRPS is important and beneficial. As a complementary treatment, exercise including graded motor imagery, progressive stress loading training, and aerobic training can improve patients’ pain and disabilities. This article briefly summarized the effectiveness of different types of exercise on CRPS and indicated the underlying mechanisms of the alleviating effect of exercise on CRPS, including the remodeling of abnormal brain structure and brain function, the reduction in peripheral sensitization, the regulation of vasodilation and adrenaline levels, the release of endogenous opioids, and the increased anti-inflammatory cytokines. Therefore, exercise is a feasible and effective treatment for patients with CRPS to improve pain intensity, physical function, and mental health. In addition, the characteristics of capricious symptom, difficult diagnosis, and refractory of CRPS indicate the need for the matched graded multi-professional care. Some complementary treatments such as medications, transcutaneous electrical nerve stimulation (Sutbeyaz et al., 2005; Anandkumar and Manivasagam, 2014; Bilgili et al., 2016), acupuncture (Peng et al., 2018), and manual lymphatic drainage (Duman et al., 2009), have also shown great effect to improve pain and oedema in combination with exercise therapy (Melf-Marzi et al., 2022). Given the comprehensive search, we found a lack of sufficient high- quality clinical studies about CRPS to carry out deeper analyses, which is one of the limitations of this study. Besides, direct evidence of neurophysiological changes in CRPS patients is also unclear. Thus, more clinical studies with larger sample size and detailed protocol in the future could provide more valuable insights. The parameters of different exercise therapy for different stages of CRPS and the integration of exercise therapy and other methods still need further exploration.

## Author contributions

X-QW and XS conceived the review. T-SL and RW searched the literature to identify eligible studies and drafted the manuscript and extracted related information. T-SL made the figure and table. All authors contributed to the revision of manuscript, and they have read and approved the final version of the manuscript and the order of presentation of the authors.

## Funding

The study was supported by the Science and Technology Commission of Shanghai Municipality [grant numbers 19080503100 and 21S31902400], the Fok Ying-Tong Education Foundation of China [grant number 161092], the Talent Development Fund of Shanghai Municipal [grant number 2021081], the Shanghai Clinical Research Center for Rehabilitation Medicine [grant number 21MC1930200], and the Shanghai Key Lab of Human Performance (Shanghai University of Sport) [grant number 11DZ2261100], as well as by the Shanghai Frontiers Science Research Base of Exercise and Metabolic Health.

## Conflict of interest

The authors declare that the research was conducted in the absence of any commercial or financial relationships that could be construed as a potential conflict of interest.

## Publisher’s note

All claims expressed in this article are solely those of the authors and do not necessarily represent those of their affiliated organizations, or those of the publisher, the editors and the reviewers. Any product that may be evaluated in this article, or claim that may be made by its manufacturer, is not guaranteed or endorsed by the publisher.
